# Target Site Insensitivity Detection in Deltamethrin Resistant *Culex pipiens* Complex in Iran

**Published:** 2019-06

**Authors:** Reza ZEIDABADINEZHAD, Hassan VATANDOOST, Mohammad Reza ABAI, Navid DINPARAST DJADID, Abbasali RAZ, Mohammad Mahdi SEDAGHAT, Mohamad Ali OSHAGHI, Ahmad RAEISI, Neda ADIBI

**Affiliations:** 1. Department of Medical Entomology and Vector Control, School of Public Health, Tehran University of Medical Sciences, Tehran, Iran; 2. Department of Environmental Chemical Pollutants and Pesticides, Institute for Environmental Research, Tehran University of Medical Sciences, Tehran, Iran; 3. Malaria and Vector Research Group, Biotechnology Research Center, Pasteur Institute, Tehran, Iran; 4. Malaria Control Unit, Center for Communicable Diseases Control, Ministry of Health and Medical Education, Tehran, Iran

**Keywords:** *Culex pipiens*, Knockdown resistance, Deltamethrin

## Abstract

**Background:**

Some mosquito species which belong to the *Culex. pipiens* complex are primary vectors for West Nile virus, Sindbis, *Dirofilaria immitis*, and many arboviruses. Knockdown resistance (kdr) mutations in the voltage-gated sodium channel (VGSC) gene of *Cx. pipiens* that is inherited, is one of the important threats for the efficacy of pyrethroids insecticides. Knockdown resistance (kdr) mutation, L1014F, is a well-defined mechanism of resistance to pyrethroids and DDT in many insect species. The aim of study was to determine the mechanisms of Insecticide resistance in this species

**Methods:**

Specimens of *Cx. pipiens*, the major vector of West Nile virus, were obtained in Tehran, Iran by collecting larvae from polluted wastewater in Qarchak of Tehran. In 2016 Insecticide susceptibility tests were performed according to WHO methods with deltamethrin 0.05%. We focused on determination of this point mutation in the VGSC gene of *Cx. pipiens* by Real-time PCR.

**Results:**

Our results revealed high levels of resistance to deltamethrin 0.05%. The lethal times i.e. LT_50_ and LT_90_ for deltamethrin were 2.1530 and 8.5117 h respectively. The result of Real-time PCR confirmed the presence of resistant genotype in all the members of tested population. This study is the first report on kdr genotyping of *Cx. pipiens* from Tehran and our results on the VGSC gene in position L1014F confirmed the TTA to TTT nucleotide change.

**Conclusion:**

This finding will provide a clue for management of insecticide resistance in mosquito which are vectors of arboviruses and decision for replacement of novel approach for vector control.

## Introduction

The global strategy determined by WHO in 1993, recommended international management of the disease, including selective vector control (1). *Culex pipiens* is considered as an important vector of a wide variety of pathogens and parasites of medical and veterinary diseases worldwide (2). Insecticides have played a central role for control of the major insect vectors of infectious diseases such as malaria, filariasis, and hemorrhagic fever since the early 20th century (3). Pyrethroids are currently considered to account for approximately 15% of the world insecticide market and are used to control many agricultural pests and disease vectors (4). Unfortunately, natural selection of resistance in vector populations has happened for every class of insecticides, which has exclusive effect on their effectiveness (5). Basic studies on insecticides have revealed that pyrethroids exert their toxic effects on the central and peripheral nervous system of insects by interacting with the voltage-gated sodium channel (VGSC) and increasing its sensitivity to depolarization by inhibiting the deactivation processes primarily (6). However, long-term use of pyrethroids, and the similarly acting DDT has been resulted in natural selection of insects with reduced target-site sensitivity to these insecticides known as ‘knockdown resistance (kdr) (7).

similarly acting DDT and deltamethrin alter the normal function of the VGSC, resulting in lengthen channel opening which causes increased nerve infuse transmission which leads to the paralytic and death (8). Resistance to DDT and deltamethrin is often affiliated with mutations in the sodium channel gene that cause decrease neuronal sensibility to these insecticides (9). Kdr or kdr-like mechanisms results from a Lucien to phenylalanine (L1014F) replacement in trans membrane segment 6 of domain II of the sodium channel (10). There are two major mechanisms of pyrethroid resistance in insects increment the rate of metabolic detoxification of insecticide, or changes in target site sensitivity caused the kdr mutation (11). Insecticide resistance management needs quick high throughput investigation to detect the resistance-associated mutations. Currently, there are several different methods accessible for detecting the accountable genetic changes for kdr in mosquitoes. The most broadly used method is allele-specific PCR (AS-PCR) (12, 2). Standard oligonucleotide primers are used in HRM (High-Resolution Melting), there is no requirement for fluorescently labelled oligonucleotides and the running costs are very low (13). In addition, HRM is generally used to identify or categories the unknown mutations in the region of DNA encompassed by the PCR primers, as any alternative base change will alter the melting profile of the amplicon (14).

This study aimed to investigate the level of susceptibility of *Cx. pipiens* mosquitoes to the deltamethrin 5% insecticide by WHO standard method and determining the molecular mechanism of deltamethrin insecticide in this type of mosquito. The kdr mutation is one of the main reasons for this type of mosquito to be resistant to insecticides.

## Methods

### Mosquito collection and insecticide susceptibility assay

All mosquito larvae samples were collected from various locations of Qarchak County including Mohammad-Abad (35° 26′ 12.270′N, 51° 34′ 20.70″ 945E) and Valiasr Industrial park (35° 26′ 12.10″N, 51° 34′ 20.70″ 936E) in 2016. Sampling was conducted from polluted wastewater containers in urban areas. Insecticide susceptibility tests were conducted according to WHO standard methods (15). Susceptibility tests were performed on the 2–3 d old female adult mosquitoes were fed by 10% solution of sugar in logarithmic time intervals. Adult females were exposed to 0.05% deltamethrin (with contact time 0.5 to 8 h in 10 replicates), in standard WHO test tubes. Control mosquitoes were exposed to paper without insecticide. According to the current WHO criteria, mortality less than 90% after one-hour exposure and 24 hour recovery period indicate the resistant. According to the current status of resistant in *Culex* the concentration and exposure times were as follow: DDT4% four hours, fenitrothion 1% two hours, deltamethrin 0.025% one hour, deltamethrin 0.05% half-hour, lambdacyhalothrin 0.025% one hour, lambdacyhalothrin 0.05% half-hour, permethrin 0.25% three hours, permethrin 0.75% one hour. After bioassay, dead (susceptible) and alive (resistant) mosquitoes were collected and stored separately at −20 °C, and were transferred to the laboratory for molecular study (16).

### PCR template preparation

Genomic DNA from 100 individual mosquitoes was extracted using High Pure PCR Template Preparation kit (MBST Company, Iran). Individual mosquitoes were homogenized in a mixture of 50 μL tissue lysis buffer kit (MBST) and the single mosquitoes were lysed in 130 μl lysis buffer and 20 μl proteinase K (10 mg/ml)) for 20 min-2 h at 55 °C. After adding 360 μl binding buffer and incubation for 10 min at 70 °C, 270 μl ethanol (96%–100%) was added to the solution. Then, it was vortexed and the complete volume was transferred to the MBST-column. The MBST-column was first centrifuged at 8000×g and then washed twice with 500 μl washing-buffer at 8000×g. The columns were then centrifuged with 12000×g to remove the rest of ethanol from the carrier. After that, the DNA was eluted from the carrier with 100 μl elution buffer. Finally, the DNA was visualized on 0.8% agarose gel in and UV-transilluminator (17).

### Primer designing

Primer designing was performed according to the submitted sequences of *Cx. pipiens* to the Gene Bank to characterize the gene region encoding domain II S6 of the sodium channel (VGSC) in Iranian species. In a range of insect species, there is an intron very close to the kdr mutation site. In many insect species, this intron shows a degree of variations (nucleotide substitutions or insertions/deletions) between different stains/isolates which would affect the performance of any assay that primer binding sites are considered within this region. Therefore, nucleotide alignments of all the *Cx. pipiens* domain II S6 sodium channel gene sequences available in the National Center for Biotechnology Information (NCBI) database was performed and two regions around the kdr site conserved in all isolates/species were selected for primer designing (Table 1).

**Table 1:** Primers used for PCR amplification of VGSC gene fragment

***PRIMER***	***primer Sequence (5′ - > 3) Se***
F116	TCTTCTTGGCCACCGTAGTGATAG
R317	GTATGAACTGTTTGTTTACATCAAAGGTACAAATG

### Real-time PCR (HRM) for kdr Detection

Based on the mentioned parameters, its advantages and performed studies in other cases, we used Real-time PCR for genotyping the kdr allele that is more sensitive and takes relatively less time. In HRM analyses a small region of DNA containing the mutation of interest is amplified by PCR in the presence of Eva green, the third generation of intercalating fluorescence dyes. The design of an HRM assay for kdr detection followed the recommendations in previous reports of this technique. The kdr-Forward and kdr-Reverse primers were used for HRM as they efficiently amplified a small product of 153 bp. PCR reactions contained 1 μl of genomic DNA, 0.5μl Primer forward, 0.5μl Primer Reverse, 3 μl Evagreen-Qpcr Mix Plus (Pishgam Company, Iran) 15 μl H2O PCR grade and 20 μl total Samples were run on a Rotor-Gene 6000 (HRM)™ using temperature cycling conditions of; Initial denaturation 15 min at 95 °C followed by 1 cycles, denaturation15 sec at 95 °C and Annealing 20 sec at 60 °C and Elongation 20 sec at 72 °C followed by 40 cycles. The increase in Eva green fluorescence was monitored in real time during the PCR and the subsequent decrease during the melt phase by acquiring each cycle/step to the green channel (470 nm excitation and 510 nm emission) of the Rotor-Gene. Genotypes were scored by examining normalized and difference melt plots using the Rotor-Gene Software.

## Results

**Fig. 1:**
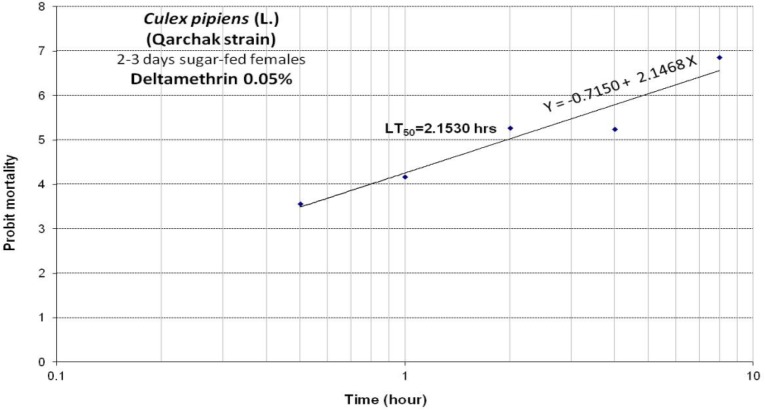
Equation, regression line and lethal time (LT_50_) of 2–3 d sugar-fed females *Cx. pipiens* exposed to Deltamethrin 0.05% collected from Qarchak County, Tehran, Iran, 2015–16

The susceptibility rate of *Cx. pipiens* mosquitoes from Qarchak southern regions of Tehran tested with deltamethrin 0.05% papers. The result of susceptibility test for filed strains of *Cx. pipiens* has been summarized in Tables 2, 3 and Fig. 1.

**Table 2:** Susceptibility levels of the field strain of *Culex pipiens* to deltamethrin 0.05%

***Exposure time (hour)***	***Results (treated group)***					***Observed probit mortality***	***Expected probit mortality***	***Results (Control group)***				
	***Replicate***	***No. Alive***	***No. Dead***	***Total***	***Mortality (%)***			***Replicate***	***No. Alive***	***No. Dead***	***Total***	***Mortality (%)***
0.5	10	221	18	239	7.5	3.562	3.639	2	49	1	50	2.0
1.0	10	218	56	274	20.4	4.174	4.285	2	48	1	49	2.0
2.0	10	111	173	284	60.9	5.277	4.931	2	49	0	49	0.0
4.0	10	111	164	275	59.6	5.244	5.578	2	45	0	45	0.0
8.0	4	3	94	97	96.9	6.868	6.224	1	25	0	25	0.0

**Table 3:** Lethal time (LT_50_ and LT_90_) values for *Culex pipiens*, Qarchak County, Tehran, Iran, 2015–16

***a***	***b ± SE***	***LT_50_ (hrs.) ± 95%C.L.***	***LT_90_ (hrs.) ± 95%C.L.***	***χ^2^ (Heterogeneity)***	***χ^2^ table (df)***	***P-value***
−0.7150	2.1468 ± 0.499	1.0702	3.8578	49.702^*^	11.345 (3)	0.01
		2.1530	8.5117			
		6.3203	768.7028			

* Heterogeneity

A= y-intercept, B= the slope of the line, SE= Standard error, CI= confidence interval, x2=heterogeneity about the regression line, df=degree of freedom, *P*>0.05=represent no heterogeneity in the population of tested mosquitos

### Molecular detection of kdr mutation

**Fig. 2:**
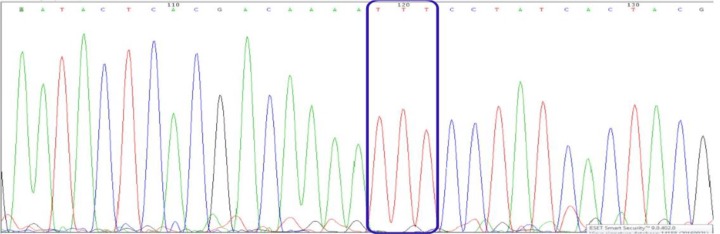
Chromatogram of VGSC gene sequence shows L1014F mutation (TTA to TTT) in deltamethrin resistant strain of *Cx. pipiens*, Qarchak County, Tehran, Iran, 2015–16

**Fig. 3:**
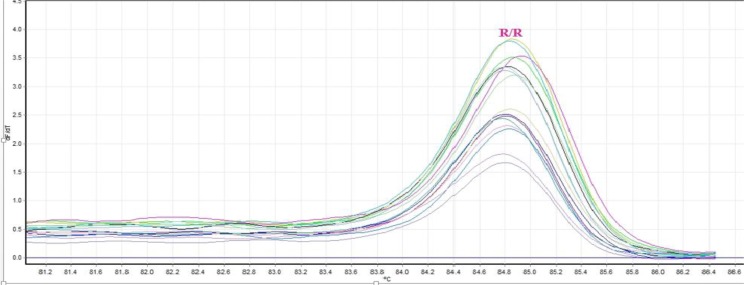
High-Resolution Melt (HRM) for detection of kdr mutation in *Cx. pipiens*

HRM analysis of the samples revealed that all of tested samples had the same genotype. To confirm real-time PCR results, 20 samples were selected randomly and sequenced. Analysis of the chromatogram confirmed their genotype similarity and revealed the A to T nucleotide substitution on kdr allele of VGSC gene in samples that were resistant to 0.05% deltamethrin (Fig. 2). Melting curve analysis of the kdr allele obtained with HRM real-time PCR for the mutant strains with the (L1014F) mutation. All the samples had the same melting profile. The mosquitoes were mutant for knockdown-resistant allele (TTT/TTT) (Fig. 3).

## Discussion

In this study, we analyzed the kdr mutation by Real-Time PCR assay followed by partial sequencing of sodium channel gene in *Cx. pipiens*. We analyzed a polymorphic site at position 1014 (TTA to TTT) in the IIS6 domain of VGSC, which induces a change in leucine to phenylala-nine in the same species The sequences found in the one strains were identical (including the region of alternative splicing) except for one codon: the non-silent replacement of TTA (leucine) present in the susceptible strain by TTT (phenylalanine) in the resistant strain as previously reported by David Martinez and Corbel (18, 19).

According to the current WHO criteria of resistance to different insecticides, we also demonstrated the high resistance of deltamethrin 0.05% insecticides. In a study in southeastern Iran, mortality rate for 0.05% deltamethrin was 93% with1 hour exposure time (20). In Urmia County northwestern Iran, determination mortality rate in *Cx. pipiens* for Deltamethrin 0.05% was 81.21% with Discriminating concentration, 1 h exposure period (21). In south Tehran, mortality rate of *Cx. pipiens* were reported 18% for deltamethrin 0.05% and LT50 for deltamethrin 0.05% was 3-h (22). In Turkey, mortality rate in *Cx. pipiens* for 0.05% deltamethrin in different area with five different strain were 95.8%, 95%, 97.5%, 80%, 75.8% (23). In the present study, the LT_50_ and LT_90_ values were 2.1530, 8.5117 for deltamethrin 0.05%, respectively.

Evaluation of the kdr mutation in *Cx. pipiens* Pallens mosquitoes in China revealed the presence of L1014F and L1014S mutations in para-sodium channel gene which were single mutations (TTA to TTT and TTA to TCA) (23). In another study, a 521-bp amplicon of DNA sequence from individual mosquitoes was amplified from 622 specimens of *Cx. pipiens* pallens using PCR. TTA is the wild-type codon sequence in the 1014 site of the sodium channel gene II S6. DNA sequence analysis revealed the presence of two point mutations at this region: L1014F (TTA1014) which replaces L with F and L1014S (TTA014S) which replaces L with S (24). In another study analysis of the kdr mutation by PCR assay was performed and followed by partial sequencing of sodium channel gene in *Cx. quinquefasciatus.* There is some similarity related to our results (25, 26, 27). First report of L1014F- kdr mutation in *Cx. pipiens* complex from Morocco showed that all specimens of susceptible mosquitoes had 1014 L/1014 L genotype (28).

Alignment of sequences *Anopheles stephensi* from the Dubai-R and Beech strains showed an A to T substitution in the open reading frame of the resistant compared to the susceptible strain, which resulted in a leucine (TTA) to phenylalanine (TTT) substitution in the permethrin-resistant strain (29).

Our HRM assay was designed to include *Cx. pipiens*. Although sequencing is still considered as the gold standard for genotyping, HRM curve analysis offers a quick and cost-effective method for genotyping the known and identification of unknown mutations. With the advent of new saturating dyes, such as Eva Green, dye concentrations can be increased without concerns of interfering with polymerase reaction. In this study, the performance of the HRM was evaluated on individual mosquitoes for genotyping the L1014F locus. Managing insecticide resistance is complex because resistance takes a variety of forms. Therefore, local strategies must be tailored to the type of resistance present. The two main mechanisms metabolic resistance (30) and target-site resistance (31) include multiple forms (32), which are of varying importance for different classes of insecticide. A further complication is ‘cross-resistance’ between insecticides that have the same mode of action for killing mosquitoes. For example, vectors that are resistant to pyrethroids and have kdr target-site resistance will probably also be resistant to DDT. Cross-resistance restricts the choice of alternative insecticide available for resistance management. Several strategies exist for IRM for vector control, based on the use of IRS and LLINs. They include rotations of insecticides, use of interventions in combination, and mosaic spraying. Potential future strategies include use of mixtures. In some settings, resistance management strategies may be implemented in the broad context of integrated vector management. These strategies can have several effects on populations of resistant vectors: they can delay the emergence of resistance by removing selection pressure (e.g. rotations) or kill resistant vectors by exposing them to multiple insecticides (e.g. mixtures, when they become available).

## Conclusion

The status of *Cx. pipiens* insecticide resistance in the urban areas of Tehran is particularly worrying. The data of our experiment confirm the presence of kdr allele in the wild population of *Cx. pipiens* in southern of Tehran. However, the exact role of this kdr mutation in development of insecticide resistance should be defined by further investigation. However, kdr is closely related to insecticide resistance and considered as an effective and apparent marker. The discovery of kdr mutation in *Cx. pipiens* from Tehran is of great significance at both fundamental and applied levels.

## Ethical considerations

Ethical issues (Including plagiarism, informed consent, misconduct, data fabrication and/or falsification, double publication and/or submission, redundancy, etc.) have been completely observed by the authors.
